# An engineered biomaterial to harness the differentiation potential of endogenous human gingival mesenchymal stem cells (hGMSCs)

**DOI:** 10.3389/fdmed.2023.1235096

**Published:** 2023-07-26

**Authors:** Mohammad Mahdi Hasani-Sadrabadi, Weihao Yuan, Sevda Sevari, Bo Yu, Sahar Ansari, Alireza Moshaverinia

**Affiliations:** ^1^ Department of Bioengineering, University of California, Los Angeles, Los Angeles, CA, United States; ^2^ Weintraub Center for Reconstructive Biotechnology, Section of Prosthodontics, School of Dentistry, University of California, Los Angeles, Los Angeles, CA, United States; ^3^ Section of Restorative Dentistry, School of Dentistry, University of California, Los Angeles, Los Angeles, CA, United States; ^4^ California NanoSystems Institute (CNSI), University of California, Los Angeles, Los Angeles, CA, United States

**Keywords:** poly(ε-caprolactone), artificial stem cell niches, autotherapy, human gingival mesenchymal stem cells (hGMSCs), polydopamine (PDA), SDF-1α

## Abstract

Here, we developed a stromal cell-derived factor-1a (SDF-1α) delivery biomaterial as an artificial polymeric-based niche with the ability to recruit local endogenous human gingival mesenchymal stem cells (hGMSCs) for craniofacial bone regeneration applications. Polydopamine-coated poly(ε-caprolactone) (PCL)-gelatin electrospun membranes were loaded with stromal cell-derived factor-1α (SDF-1α) via physical adsorption. Subsequently, the release profile of SDF-1α and the chemotactic capacity on human bone marrow mesenchymal stem cells (hBMMSCs) and hGMSCs were evaluated. The osteogenic differentiation capacity of the recruited MSCs was also assessed *in vitro*. Our results confirmed the sustainable release of SDF-1α from the developed biomaterial promoting the migration and homing of human bone marrow mesenchymal stem cells (hBMMSCs) and hGMSCs. Moreover, the results of the osteogenic differentiation assay showed that SDF-1α delivery significantly enhanced osteogenic differentiation of hBMMSCs and hGMSCs and up-regulated the gene expression of osteogenic markers compared to the control group. In conclusion, the current study successfully developed a novel and effective treatment modality for craniofacial bone regeneration by recruiting the autogenous progenitor cells including hGMSCs. The developed niches can potentially lead to the development of a novel platform for targeted manipulation of *in vivo* microenvironment to achieve efficient and safe craniofacial cell reprogramming, which also will pave the road to determine the capacity of local hGMSCs' contribution to *in situ* bone regeneration.

## 1. Introduction

In the US, over a million bone reconstructive procedures are performed due to injury, surgical removal of diseased tissue, or congenital defects, representing substantial morbidity, pain, and disability tolls on society (1–3). Also, this induces major social and economic hurdles, as bone regeneration therapies represent a cost of more than $2.5 billion in the US each year. Clinicians are often faced with a challenging task of harvesting and grafting to recreate the necessary tissue architecture and function (4). Recent progress in using stem cells for tissue regeneration has raised some challenges including invasive harvesting procedures, acquisition of adequate cell numbers, impaired *in vivo* differentiation efficiency, along with lower genetic stability of the *ex vivo* expanded stem cells (5, 6). Despite all the promising attempts, a big portion of the engrafted cells are lost immediately post-implantation due to several stresses that cells encounter from the microenvironment. Therefore, an alternative stem cell-based bone regenerative therapy could utilize the endogenous healing capability of the local progenitor stem cells residing in the postnatal oral tissue to avoid the injection of exogenously manipulated cells (7, 8). Autotherapy is a type of therapy in which an individual is treated with their own bodily substances, which can activate the body's natural healing mechanisms (7–10). Specifically, autologous stem cell therapy involves the use of an individual's own stem cells to promote tissue regeneration and repair. The stem cells, e.g., GMSCs, can be obtained from the patient through minimally invasive procedures and are processed and prepared for use. Development of biomaterials as artificial niches presents a promising approach to the recruitment of endogenous progenitor cells via presenting different physical or biochemical signals (9, 10). Subsequently, an artificial niche containing bioactive/signaling molecules can be constructed that has the capability *to* recruit endogenous cells stimulating the body's repair mechanisms (11–16). The physical properties and bioactivity of the modern available biomaterials can be tuned to optimize the recruitment of endogenous cells in the microenvironment. In addition to the bioactivity and cell recruitment capabilities, an ideal biomaterial for bone tissue regenerative applications should be biocompatible, exhibit a favorable degradation rate, and present no risk of disease transmission (17–20).

Human gingival tissue harbors fibroblasts that are functionally equivalent to mesenchymal stem cells (MSCs) with potential osteogenic and immunoregulatory properties (14, 21–24). The activated T cells are known to be reprogrammed secreting higher amounts of anti-inflammatory cytokines such as IL-4 and IL-10 upon interacting with fibroblasts (25, 26). Since the regenerative capacity of the human gingival mesenchymal cells (hGMSCs) is known to be controlled by their surrounding gingival ECM, it is necessary to develop a tool that allows for hGMSCs to share positively in bone regeneration. Polymeric scaffolds containing polydopamine (PDA) coatings have been generated that offer osteogenic properties (27, 28). Incorporation of stromal cell-derived factor 1 (SDF-1) has shown to recruit local progenitor cells leading to enhanced tissue regeneration (29–32). Combining these advancements to form an artificial niche for craniofacial applications has not been explored. We have previously developed a PDA-coated poly(caprolactone) (PCL)-based nanofibrous scaffold with tunable mechanical and biodegradation properties (29). Here, we utilized the developed niche as a drug delivery vehicle, for controlled release of SDF-1, as an artificial niche to recruit the local GMSCs. The nanofibrous scaffold can be sutured to the tissue and PDA coating simultaneously promotes cell adhesion and osteogenic reprogramming of the recruited GMSCs. PCL was selected as it has FDA approval for human use, and its mechanical characteristics can be tuned to match the desired native tissues. In addition to the osteogenic potential of PDA, we believe that local recruitment to the defect site could allow for the GMSCs to be subjected to the ECM components of the bony surfaces of the remaining structures, promoting their behavior in response to bone mediators that will lead to *in vivo* reprogramming (33). We predict that the reprogrammed osteoblasts could further contribute to ECM remodeling and bone regeneration through the secretion of various growth factors.

## 2. Materials and methods

### 2.1. Fabrication of polymeric-based nanofibrous scaffold

All chemicals were purchased from Sigma-Aldrich, Inc. (St. Louis, MO, USA). Cell culture reagents, solutions, and dishes were obtained from Thermo Fisher Scientific (Waltham, MA, USA). Electrospinning was used to engineer a nanofibrous scaffold based on poly(ε-caprolactone) (PCL) according to the methods published previously (29). Briefly, ester-terminated PCL polymer (10% w/w) was dissolved in hexafluoroisopropanol and electrospun using an electrospinning device at 20 kV. To obtain morphological patterning, a stainless steel metal mesh substrate was utilized.

In order to provide degradability and cell adhesion sites while providing favorable strength, gelatin (Sigma-Aldrich, from porcine skin) was added to the PCL solution (PCL: gelatin, 3:1). Polydopamine coating of the developed membranes was achieved according to our previously published protocols (29). Membranes were incubated in dopamine hydrochloride in Tris-HCl buffer at room temperature overnight. The coated membranes were washed at least three times and dried with nitrogen gas.

### 2.2. Structural and morphological characterization of engineered niches

Nanofibrous niches were characterized using scanning electron microscopy (SEM, ZEISS Supra 40VP) at an accelerating voltage of 5 kV to check morphology and the uniformity of nanofibers.

### 2.3. Incorporation and release measurement of SDF-1α into the nanofibrous scaffolds

PCL-PDA niches were incubated with SDF-1α (Sigma) at concentrations of 100, 200 or 400 ng/ml at 4°C under gentle shaking for 12 h. The kinetics of SDF-1α release from scaffolds with different composition and fiber diameters were studied using ELISA (R&D systems) at different time intervals.

### 2.4. Stem cell isolation and culture

Institutional review board (#BUA6510) approval was obtained to extract mesenchymal stem cells from human gingival tissues. Briefly, young healthy male individuals undergoing third molar extractions were selected for extraction of gingival tissues. Human GMSCs were isolated and cultured according to previously published procedures (34). GMSCs and hBMMSCs were separately cultured in a regular culture media containing alpha-MEM (Invitrogen) with 15% FBS, 2 mM L-glutamine (Invitrogen), 100 nM dexamethasone, 0.5 mM ascorbic acid (Sigma), 2 mM sodium pyruvate (R&D Systems Inc, Minneapolis, MN), 100 U/ml penicillin, and 100 lg/ml streptomycin (Sigma). Cells with passage 4 were used in the experiments.

### 2.5. Examination of cytocompatibility

To examine the biocompatibility of the engineered scaffolds *in vitro*, 6 mm disks of the synthesized scaffolds were prepared and placed in 48-well plates and seeded with hGMSCs (2 × 10^4^ cells/well). Cellular viability and metabolic activity were measured over 7 days using a Live/Dead Assay Kit (Invitrogen).

### 2.6. Migration tests

To assess the biological function of the released proteins, a Costar transwell system (Fisher Scientific) was used to investigate the effectiveness of the released factor on the recruitment of hGMSCs. Briefly, disc-shaped specimens (diameter 15 mm) of developed PCL/gelatin nanofibrous scaffolds with and without SDF-1α (400 ng/ml) were placed at the bottom chamber containing 1 ml of regular culture media, 1 × 10^5^ of hGMSCs or hBMMSCs were cultured in the upper chamber, and the migration rate of the cells was conducted over 48 h of culturing at 37°C and 5% CO2. The migrated cells were visualized after they were fixed in 10% formalin and stained in crystal violet (0.05%).

### 2.7. Osteogenic assay

SDF-1α containing membranes (400 ng/ml) were placed into 24-well plates. Then, either hGMSCs or hBMMSCs were plated onto the SDF-1α containing membranes. On day 1, the complete medium was changed to osteogenic medium supplemented with 10 nM dexamethasone (DEX), 10 mM β-glycerophosphate sodium, and 0.5 mM ascorbic acid (AA) (Sigma-Aldrich, St. Louis, MO). The OM was changed twice per week. After four weeks of culturing in the osteogenic media (OM), xylenol orange, a fluorescent probe that chelates to calcium and stains mineral red, was used for osteogenic characterization.

Additionally, after 2 weeks of culturing in osteogenic media, RNA was extracted from the specimens. according to the manufacturer's recommendations. Single-stranded cDNA synthesis was performed with 100-ng total RNA using a SuperScript III cDNA synthesis kit (Invitrogen). The relative production of each mRNA was determined and normalized to the expression of the internal housekeeping gene GAPDH. Primer and probe sequences are described in Table 1. PCR products were subjected to 1% agarose gel electrophoresis with ethidium bromide staining and visualized under ultra violet light illumination. SDF-1α-free scaffolds without cells were used as the negative control in this study.

**Table 1:** Primers used in PCR analysis.

Gene	Sequence	Product (bp)
*Runt-related transcription factor 2 (Runx 2)*	Sense 5′-CAGTTCCCAAGCATTTCATCC-3′	289
	Antisense 5′-TCAATATGGTCGCCAAA CAG-3′	
*Osteocalcin (OCN)*	Sense 5′-CGTGGTGACAAGGGTGAGAC-3′	292
	Antisense 5′-TAGGTGATGTTCTGGGAGGC-3′	
*Glyceraldehyde 3-phosphate dehydrogenase (GADPH)*	Sense 5′-AGCCGCATCTTCTTTTGCGTC-3′	418
	Antisense 5′-TCATATTTGGCAGGTTTTT CT-3′	

### 2.8. Statistical analysis

Data are presented as mean values ± standard deviation. Data were statistically analyzed using a one-way analysis of variance (ANOVA) or Student's *t*-test with a significance level of *p* = 0.05: *p < 0.05, **p < 0.01.

## 3. Results and discussion

### 3.1. Incorporation of SDF-1α on developed nanofibrous scaffolds

**Figure 1:**
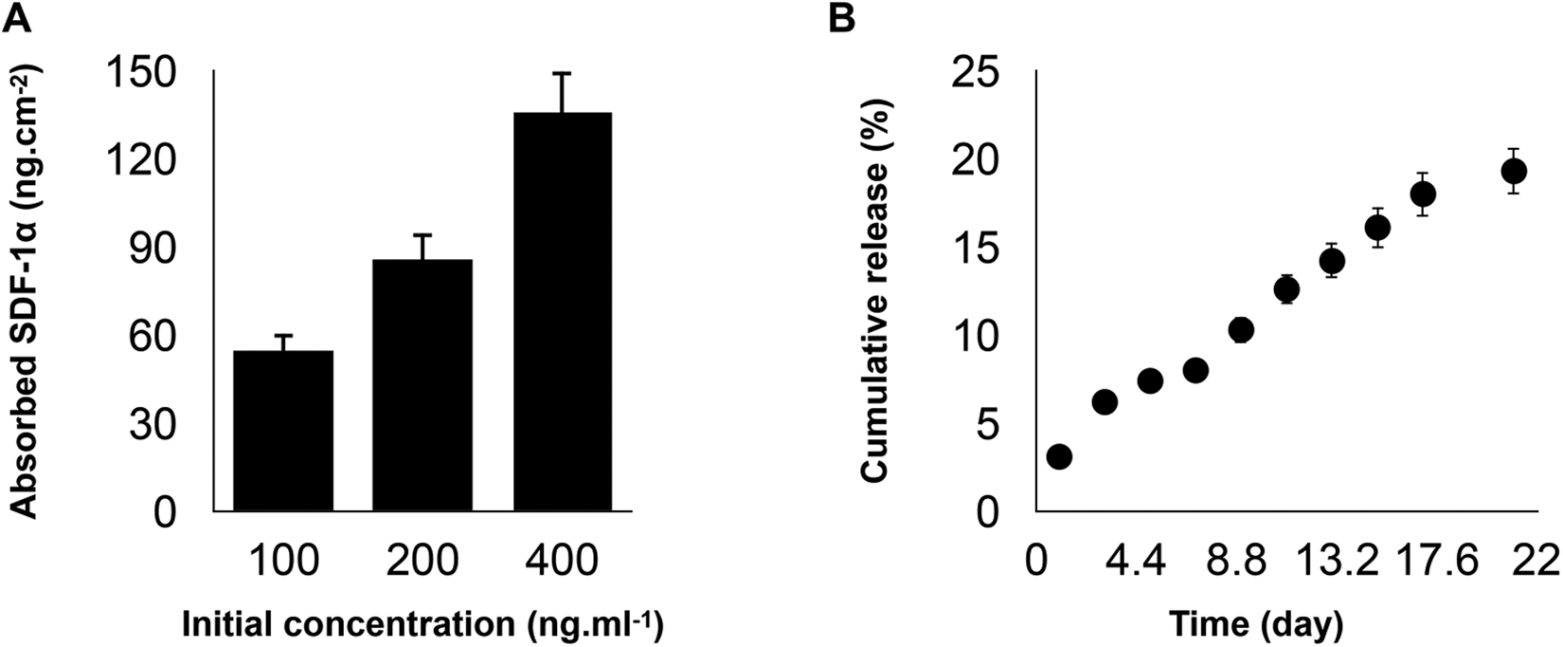
(**A**) Binding capacities of poly(ε-caprolactone)-polydopamine (PDA) nanofibrous scaffold were evaluated after co-incubation with stromal cell-derived factor 1 (SDF-1α) cytokine at concentrations of 100, 200, or 400 ng/ml at 4°C under gentle shaking for 12 h. (**B**) The cumulative release profiles of SDF-1α over two weeks at 37°C. For all the tests, statistical significance was observed with ***p < 0.001, *n* = 3.

Current stem cell-based therapies for bone regeneration don't provide favorable outcomes due to the limitations related to the injection of exogenous stem cells. In order to overcome this limitation, the recruitment of endogenous progenitor stem cells and utilization of the regenerative potential could be a promising alternative approach. There are several important physical and biological parameters to consider when developing an ideal biomaterial as an artificial niche including tunable mechanical properties, desirable biodegradation, and presentation of bioactive molecules/signals. We developed PDA-coated micropatterned nanofibrous membranes via electrospinning, followed by the incorporation of SDF-1. Our results showed that the adsorption of SDF-1α on the developed scaffold has a concentration-dependent binding. The incubation with 400 ng/ml of SDF-1α can achieve the highest adsorption density (Figure 1A). PDA coating provides favorable adhesion to adsorb substantial amounts of SDF-1α due to the intermolecular interactions of the primary amine and/or thiol groups of proteins with PDA surfaces and enables its prolonged release without any need for an additional carrier (Figure 1B).

### 3.2. Morphology and *in vitro* cytocompatibility assessment of engineered PDA-coated micropatterned membranes

**Figure 2:**
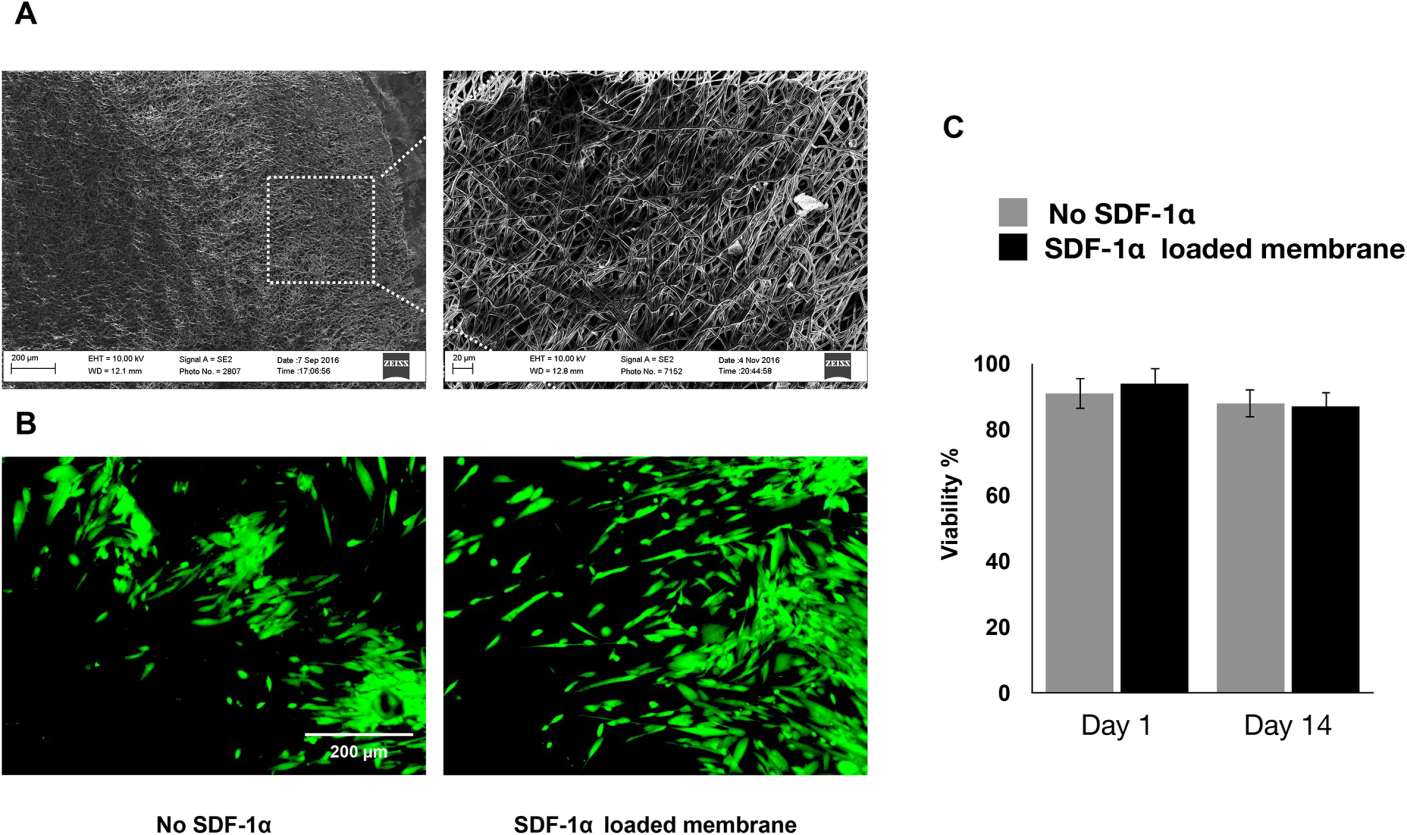
(**A**) SEM images of the membranes after two weeks of culturing in regular media. (**B**) If images showing viability of hGMSCs cultured on the engineered scaffolds after 7 days of incubation. (**C**) Quantitative live/dead results showing the viability of the cultured hGMSCs on SDF1α loaded membranes vs. membranes without SDF1α.

Engineered PDA-coated micropatterned membranes were visualized using scanning electron microscopy (SEM) to reveal their morphology. SEM images showed a highly porous structure with interconnected fibers of varying diameters and orientations. The fibers were arranged in a random organization, which significantly benefited the incorporation of SDF-1 and cell adhesion (Figure 2A). Good cytocompatibility is an essential factor to evaluate engineered niches for cell loading and tissue regeneration. We performed LIVE/DEAD staining to evaluate the cell viability of hGMSCs cultured on engineered niches. hGMSCs cultured on uncoated and SDF-1-coated membrane niches showed good adhesion and elongated morphology, demonstrating similar viability and biocompatibility of engineered PDA-coated micropatterned membranes (Figure 2B). The quantification results also suggested that the GMSCs cultured on different niches have consistent viability through the culture up to 14 days (Figure 2C).

### 3.3. Migration of hBMMSCs and GMSCs cultured on engineered PDA-coated micropatterned membranes

**Figure 3:**
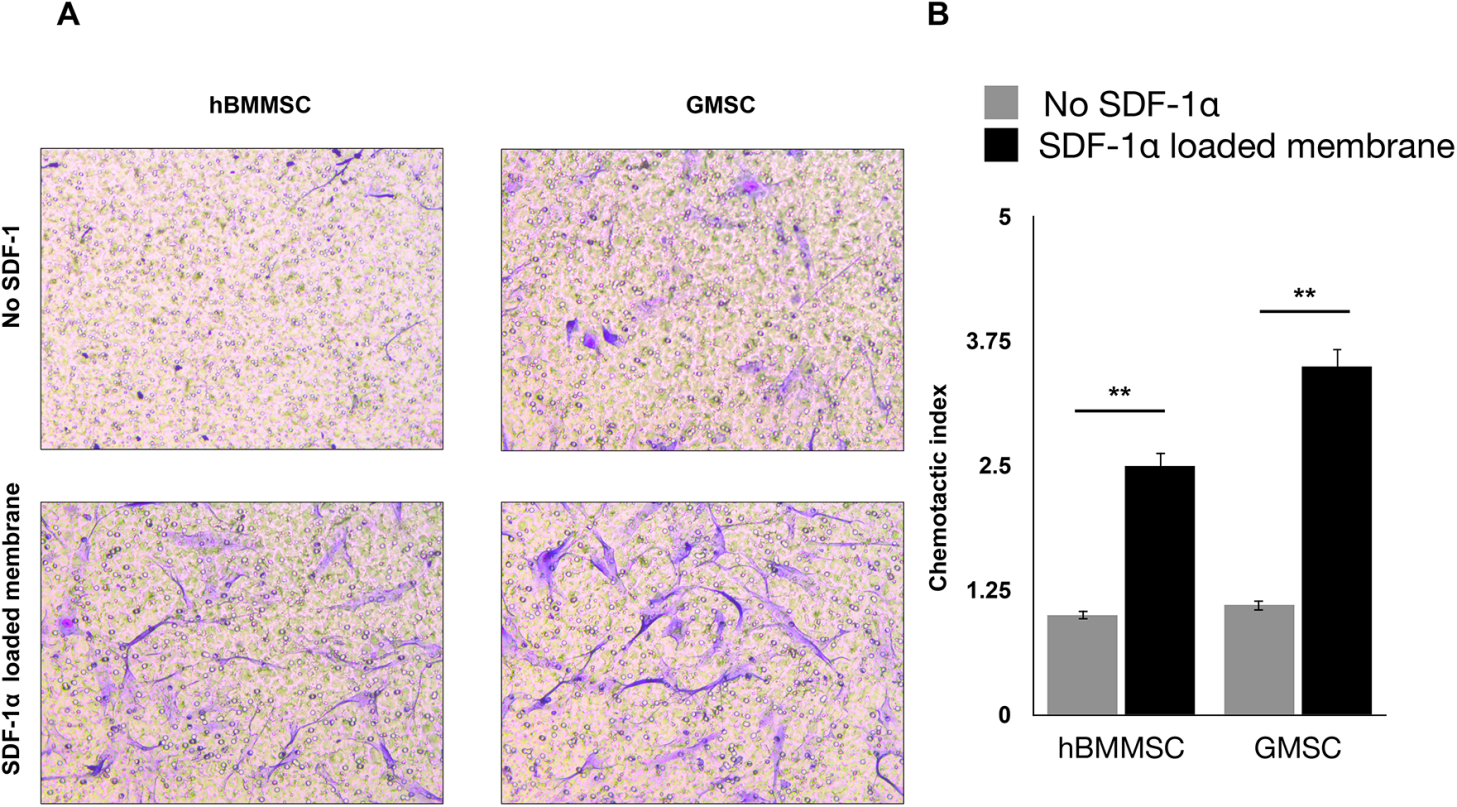
(**A**) Migration of hBMMSCs and GMSCs as studied by the transwell chamber assay. Representative images of migrated cells (stained in blue) in the lower portion of the transwell membrane after 20 h. Scale bars = 100 µm. (**B**) Quantification of relative stem cell migration (chemotactic index) for the studied groups.

Our studies showed that sustained release of SDF-1 can induce recruitment of the co-cultured hGMSCs, where the migration of hBMMSCs was used as control. The crystal violet staining of migrated cells showed that both hBMMSCs and hGMSCs have a faster migration rate when engineered niches were coated with PDA (Figure 3A). The quantification results also confirmed that GMSCs showed a better response to the SDF-1 treatment as evidenced by more stained cells on the substrates, which suggests that hGMSCs are great candidates for tissue regeneration (Figure 3B).

### 3.4. Osteogenic differentiation of hBMMSCs and GMSCs cultured on engineered PDA-coated micropatterned membranes

**Figure 4:**
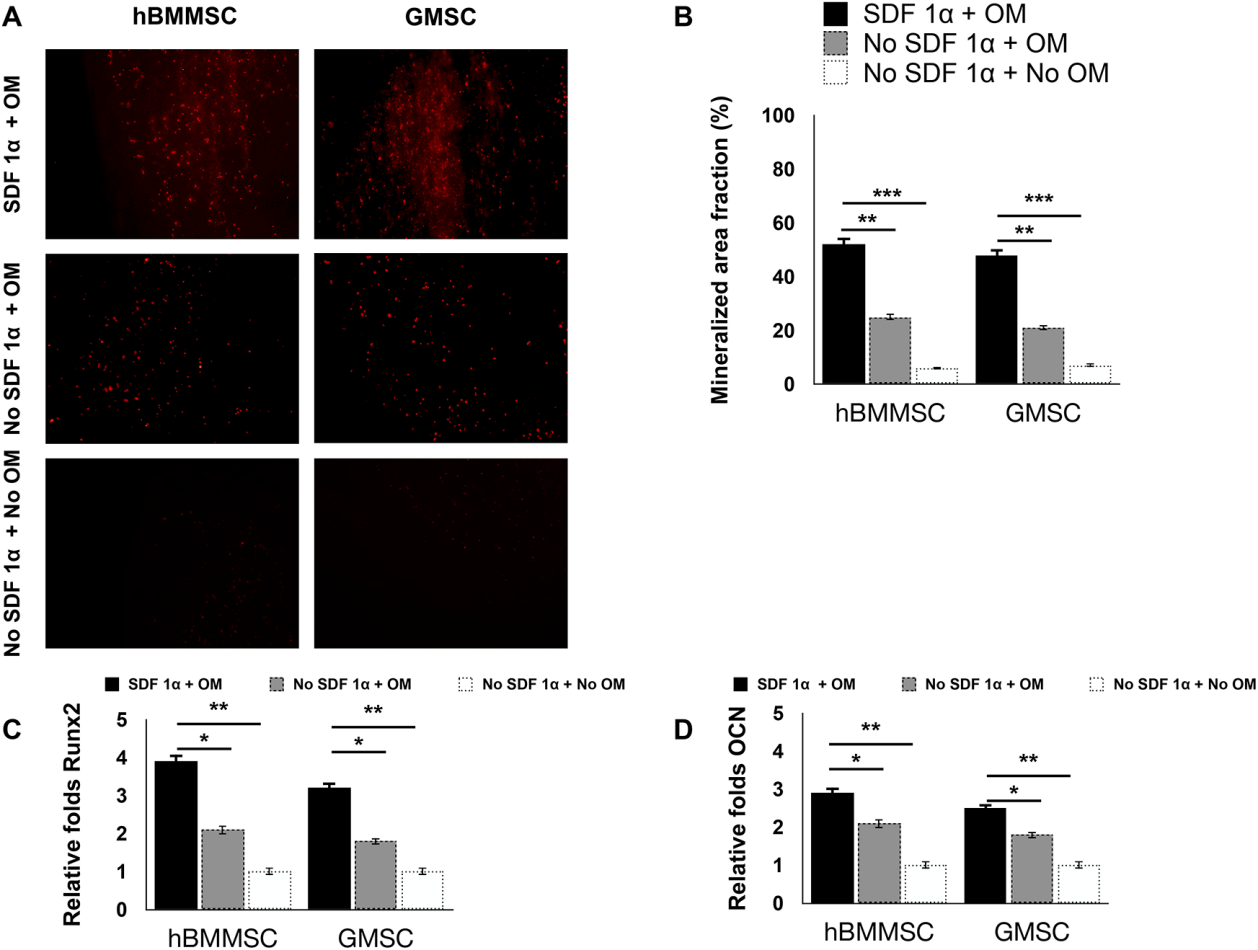
*In vitro* osteogenesis assay. PCR-based expression levels of (**A**) Runx 2 and (**B**) OCN. (**C**) Histochemical staining analysis of mineralization. Mineralization analysis of the hBMMSCs and hGMSCs after four weeks of culturing in osteogenic medium using xylenol orange. (**D**) Quantification of staining intensity of xylenol orange.

We assessed the osteogenic differentiation of hBMMSCs and hGMSCs cultured on engineered PDA-coated micropatterned membranes. Both hBMMSCs and hGMSCs cultured on SDF-1-coated membranes in an osteogenic medium (OM) showed significant osteogenic differentiation, whereas those cells cultured on uncoated membranes in OM showed modest osteogenic differentiation. The negative control group (“no SDF 1α + no OM”) showed minimal staining intensity (Figure 4C). The quantification results showed the staining intensity of xylene orange of cells in the “SDF 1α + OM” group was 2.5-fold higher than those in the “no SDF 1α + OM” group (Figure 4D). We further performed PCR tests for typical osteogenic genes, including Runt-related transcription factor 2 (Runx 2) and osteocalcin (OCN). Our results suggested that the expression levels of Runx 2 and OCN of cells in the “SDF 1α + OM” group were 2-fold and 1.5-fold higher than those in the “no SDF 1α + OM” group (Figures 4A,B).

## 4. Conclusion

We confirmed the sustained release of SDF-1α from the developed PCL/Gelatin electrospun scaffolds. Moreover, SDF-1α release enhanced the recruitment of hBMMSCs and hGMSCs and their osteogenic differentiation subsequently. It can be envisioned that the sustainable release of SDF-1α promotes the migration and homing of local endogenous hBMMSCs and hGMSCs *in vivo* improving the bone regenerative capacity of the developed scaffolds.

## Data Availability

The raw data supporting the conclusions of this article will be made available by the authors, without undue reservation.
